# Genome-Wide Characterization and Comparative Genomic Analysis of the Serpin Gene Family in Microsporidian *Nosema bombycis*

**DOI:** 10.3390/ijms24010550

**Published:** 2022-12-29

**Authors:** Maoshuang Ran, Yulian Shi, Boning Li, Heng Xiang, Meilin Tao, Xianzhi Meng, Tian Li, Chunfeng Li, Jialing Bao, Guoqing Pan, Zeyang Zhou

**Affiliations:** 1 State Key Laboratory of Silkworm Genome Biology, Southwest University, Chongqing 400715, China; 2 Chongqing Key Laboratory of Microsporidia Infection and Control, Southwest University, Chongqing 400715, China; 3 College of Animal Science and Technology, Southwest University, Chongqing 400715, China

**Keywords:** microsporidia, *Nosema bombycis*, serpins, phylogeny, expression pattern

## Abstract

Microsporidia are ubiquitous in the environment, infecting almost all invertebrates, vertebrates, and some protists. The microsporidian *Nosema bombycis* causes silkworms pébrine disease and leads to huge economic losses. Parasite secreted proteins play vital roles in pathogen–host interactions. Serine protease inhibitors (serpins), belonging to the largest and most broadly distributed protease inhibitor superfamily, are also found in Microsporidia. In this study, we characterized 19 serpins (NbSPNs) in *N. bombycis*; eight of them were predicted with signal peptides. All NbSPN proteins contain a typical conserved serpin (PF00079) domain. The comparative genomic analysis revealed that microsporidia serpins were only found in the genus *Nosema*. In addition to *N. bombycis*, a total of 34 serpins were identified in another six species of *Nosema* including *N. antheraeae* (11), *N. granulosis* (8), *Nosema* sp. *YNPr* (3), *Nosema* sp. PM-1 (3), *N. apis* (4), and *N. ceranae* (5). Serpin gene duplications in tandem obviously occurred in *Nosema antheranae*. Notably, the NbSPNs were phylogenetically clustered with serpins from the *Chordopoxvirinae*, the subfamily of Poxvirus. All 19 *NbSPN* transcripts were detected in the infected midgut and fat body, while 19 *NbSPN* genes except for *NbSPN12* were found in the transcriptome of the infected silkworm embryonic cell line BmE-SWU1. Our work paves the way for further study of serpin function in microsporidia.

## 1. Introduction

Microsporidium is a phylum of fungal-related and obligate intracellular parasitic pathogens that can invade a variety of hosts ranging from protists to insects and mammals including humans [1,2]. The first microsporidium *Nosema bombycis* was identified in silkworms by Nägeli in 1857. Since then, approximately 1700 species of microsporidia have been described in 220 genera, and new species are being discovered each year [3,4]. Transmissible microsporidia spores are ubiquitous and can infect agriculturally relevant hosts (silkworm, bees, shrimp, fish, etc.), which cause substantial economic losses [5,6]. In addition, 17 species of microsporidia have been recognized as emerging human pathogens responsible for opportunistic infections in AIDS and other immunocompromised patients [4,7]. Although over 50 microsporidia genomes have been sequenced, their molecular pathogenesis remains poorly understood [8].

Serpins are an essential superfamily of endogenous protease inhibitors with key physiological and biological roles. More than 1500 serpins have been identified in all kingdoms including plants, animals, fungi, bacteria, and viruses [9,10,11]. Most serpins inhibit serine proteases, while some serpins can inhibit cysteine proteases and papain-like cysteine proteases [12,13,14]. Despite the poor amino acid sequence homology among family members, core regions of serpins share conserved secondary and tertiary structures, usually consisting of 8–9 alpha helices, three β-sheets (A, B, C sheet), and a reactive center loop (RCL) [10]. Serpins function as suicidal protease inhibitors with unusual inhibitory mechanisms [15]. They take advantage of the energy difference between two physiologically related conformational states: (I) In the natural state, solvent exposure, and flexible RCL from the center of the β-sheet of the protein, which is similar to the typical substrate of the target protease [16,17]. The cleavage of the RCL active site by proteases leads to a conformational change to the relaxed, lower-energy-cleaved state (II), in which the cleaved RCL is inserted into the A-sheet as an additional chain. The conformational transition from stress to relaxation dynamically captures the covalent serpin–protease complex and irreversibly inhibits aggressive proteases by twisting the active site residues [18,19,20]. Recently, the study of serpin function in infection and inflammation has been of particular interest, especially as more serpins from pathogens have been identified and characterized [21]. The presence of serpins indicates their important function in pathogen survival and host interaction [22,23,24,25,26,27].

Dozens of serpins have been annotated in the *Nosema bombycis* genome, and their functions are believed to be involved in the autoregulation of proteases and the host proteases [28]. In pébrine disease, the immune responses and hemolymph melanization of the silkworm were inhibited. Previous studies have identified the serpins NbSPN6, NbSPN9, NbSPN14, and NbSPN19 transcribed persistently over the infection process, while serpins NbSPN2 and NbSPN13 are highly expressed at later stages of infection [28,29]. *Nosema bombycis* serpins may suppress the proteolysis of polyphenol oxidase (PPO) and phenoloxidase (PO), ultimately suppressing melanin formation [29]. Subsequent studies have found that NbSPN6 is expressed in the hemolymph of infected silkworms and that the recombinantly expressed NbSPN6 could inhibit silkworm hemolymph melanization. It was verified that NbSPN6 inhibited the process of PPO to PO activation by interacting with prophenoloxidase-activating enzyme (PPAE), thereby hindering hemolymph melanization [30]. Taken together, the functions of *N. bombycis* serpins have been partially characterized, but the origin and evolution of the serpin family in microsporidia are still unclear. Moreover, a comparative genome analysis has yet not been performed.

The aim of this study was to analyze the physical and chemical properties, genome locations, gene structures, and evolutionary relationships of the serpin family in *N. bombycis*, and explore serpins from other *Nosema* species. Furthermore, we obtained the expression profiles of serpins in infected silkworm tissues and BmE-SWU1 cells. Our work will lay the groundwork for future research into the functions of serpin genes in host–microsporidia interactions.

## 2. Results

### 2.1. Genome-Wide Identification of Serpin Gene Family Members

Nineteen serpin family genes were identified in the *N. bombycis* genome. These genes were named *NbSPN1–19* in the order of scaffold size. A detailed list is presented in Table 1 and Table S2. The theoretical isoelectric points (pIs) of NbSPNs ranged from 4.45 (NbSPN15) to 9.32 (NbSPN19). The lengths of the serpin genes ranged from 330 bp to 1218 bp, while the polypeptide sequences of serpins ranged from 109 to 405 amino acids, and the molecular weights ranged from 12.98 kDa (NbSPN12) to 47.96 kDa (NbSPN2). The domain analysis demonstrated that all NbSPNs possessed one serpin domain, although some of their amino acid sequence lengths were significantly shorter than the common serpin. The results of the signal peptide analysis indicate that eight of the 19 NbSPNs contained an N-terminal signal peptide, suggesting that they are likely to be secreted into the host cell.

### 2.2. Multiple Sequence Alignment and Conserved Motifs of NbSPN Proteins

**Figure 1:**
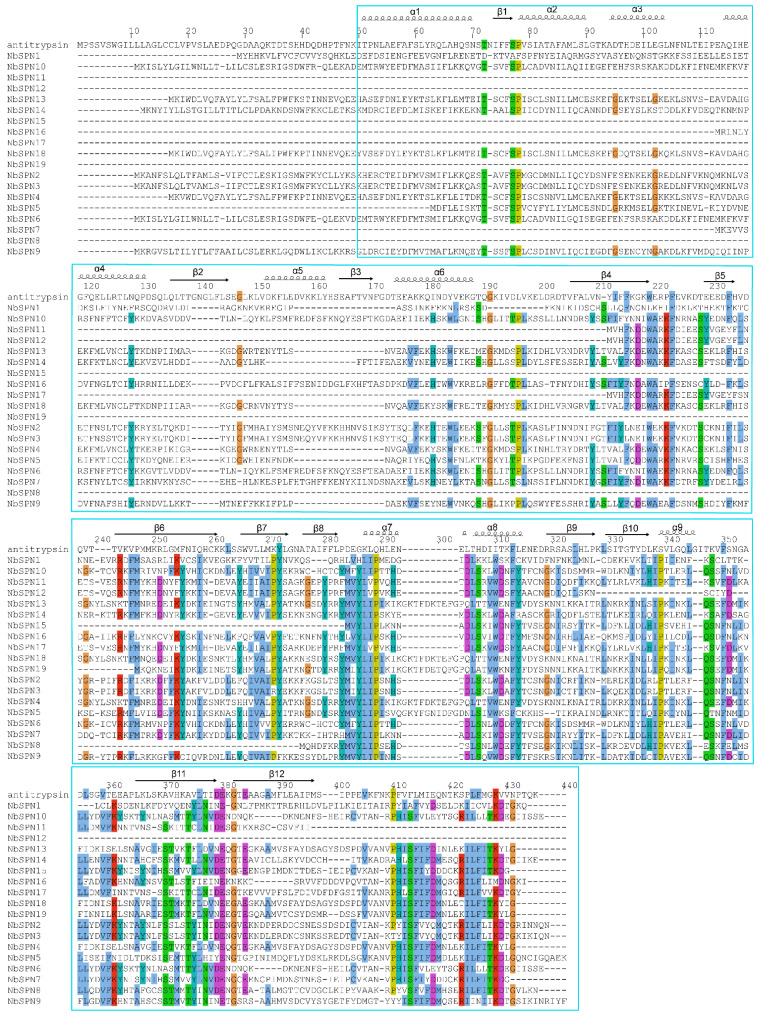
Multiple sequence alignment of all NbSPNs in *Nosema bombycis*. A blue box marks the typical conserved serpin domain. Human α1-antitrypsin (1QLP) with “template” numbering and the assignment of secondary structures of the cleaved form are included as the top sequence. The alignment of sequences was conducted with ClustalX and shaded with ESPript 3.0 online. Residues were considered as highly similar and are colored in red and framed in blue.

**Figure 2:**
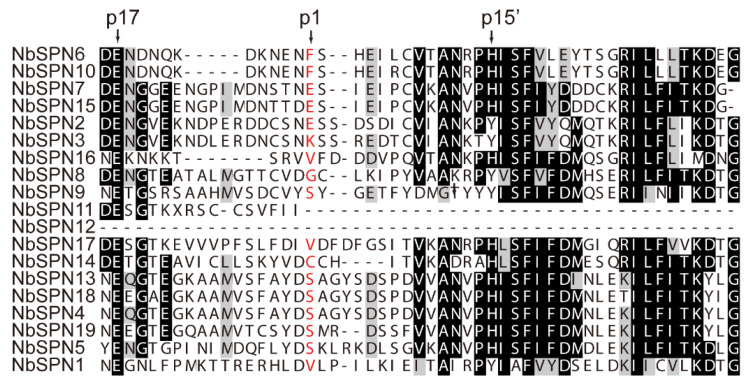
Alignment of the RCL region of *N. bombycis* serpins. The conserved residues p17 and p15′ were labelled in the hinge region of inhibitory serpins above the alignment. Predicted P1 residues are in red.

Multiple sequence alignments were performed to identify the conserved motifs of the NbSPN. The results showed a low degree of sequence similarity, but all of the proteins were found to have a serpin superfamily domain (Figure 1). Serpins can generally be recognized by a consensus pattern in their hinge regions: P17: E, P16: E/K/R, P15: G, P14: T/S, P12-P9 (A/G/S) [9,31]. These residues permit the efficient and rapid insertion of RCL into the A β-sheet. Additionally, the presence of proline in the proximal RCL before the P2 residue disrupts the formation of a new β-strand, leading to the failure of RCL incorporation [32,33]. Based on the above criteria and the alignment of the RCL region, most NbSPNs are more likely to encode flexible hinge regions, few proline residues in the proximal RCL, and highly conserved residues in the breach and shutter regions, making them more plausible to function as proteinase inhibitors, except for NbSPN11 and NbSPN12, which lack the RCL region (Figure 2).

**Figure 3:**
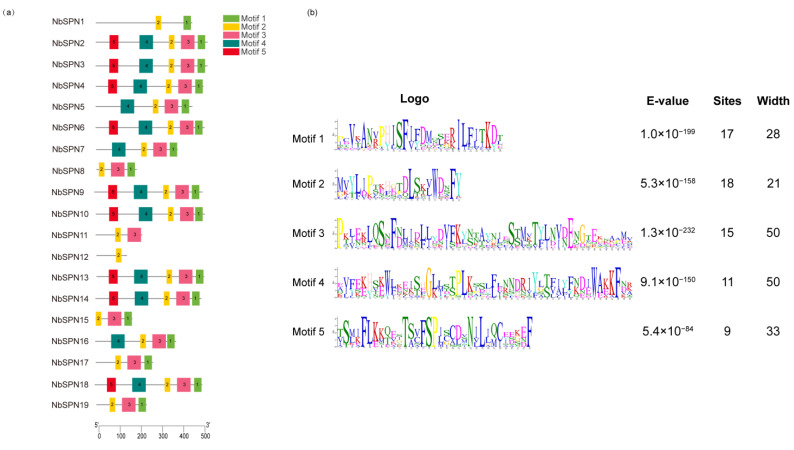
Conserved motifs of the NbSPN genes. (**a**) Distribution of conserved motifs in 19 NbSPNs, the differently colored boxes represent different bases, and the motif numbers are shown in the colored boxes. The scale represents the amino acid sequence length. (**b**) The features of the five conserved motifs among the NbSPN proteins.

In total, five conserved motifs among the NbSPNs were predicted by MEME (Figure 3), of which motif 2 was present in all NbSPNs. Motif 1 was conserved in all NbSPNs except for NbSPN11 and NbSPN12. Motif 5, which may be responsible for interacting with different target proteases, showed a high variability among the NbSPN members. These conserved motifs contribute to the conservation of the tertiary structures and functions.

### 2.3. Phylogenetic Analysis of NbSPNs

**Figure 4:**
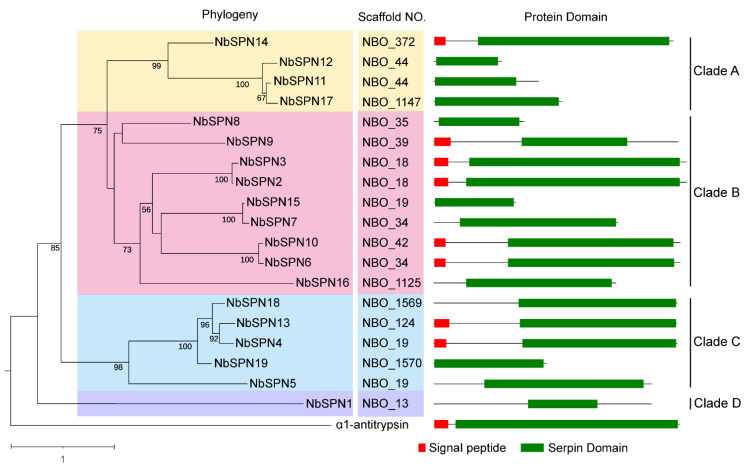
Phylogenetic analysis of NbSPNs and Protein Domain. The neighbor-joining phylogenetic tree was based on serpin protein sequences using MEGA-X. The archetypal serpin α1-antitrypsin was used as the out group. The different colors are to highlight the branches. Serpins with the predicted signal peptides (SignalP-5.0 Online: https://services.healthtech.dtu.dk/service.php?SignalP-5.0, accessed on 6 June 2022) are labeled with a red square.

The phylogenetic analysis revealed that the NbSPNs were mainly clustered into four clades. The phylogenetic tree shows that NbSPNs were relatively conserved in that all members contained the serpin domain. There were four, nine, and five members in Clades A, B, and C, respectively, eight of which had the predicted signal peptide, suggesting that these serpins could be secreted into the host cell. Clade D only had one member, NbSPN1, without the predicted signal peptide (Figure 4). Multiple sequence alignment, cluster analyses and the extremely high amino acid similarity showed duplications within the four pairs of NbSPN2-3 (Percent identity: 95.31 %), NbSPN6-10 (Percent identity: 93.92%), NbSPN4-13-18 (Percent identity: 89.49%, 89.74%, 85.38%), and NbSPN7-15 (Percent identity: 95.45%). To determine whether selective pressure acts on NbSPNs, the amino acid substitution rate between the 19 members (342 gene pairs) was calculated. The results showed that the amino acid substitution rate value ranged from 0.111 to 0.937, and the average was 0.444 (Table S3). The results showed that the synonymous substitution rate of all gene pairs was greater than the non-synonymous substitution rate (Ka/Ks < 1), suggesting that they were subjected to purifying selection and their functions were conservative.

### 2.4. Genome Distribution of NbSPNs

**Figure 5:**
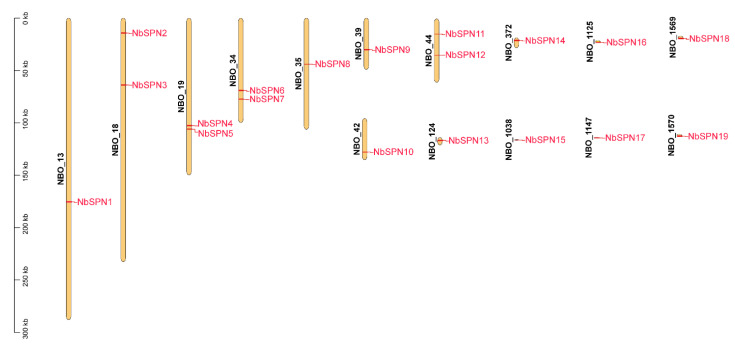
Genomic distribution of NbSPN genes across the fifteen *N. bombycis* scaffolds. The scaffold count is shown on the left side of each scaffold. The scale is in kilobase (Kb). The scaffold locations of the *NbSPNs* were determined according to the physical location of each gene.

The positions of the *NbSPNs* in the *N. bombycis* genome were analyzed. The results indicated 19 *NbSPNs* distributed on 15 scaffolds (Figure 5). The NBO_18, NBO_19, NBO_34, and NBO_44 scaffolds had two serpin genes each. *NbSPN2-3*, *NbSPN4-5*, *NbSPN6-7*, and *Nb11-12* were closely distributed on the scaffold, but not in tandem, and there was at least one gene between them.

### 2.5. Comparative Genome Analysis of the Serpin Genes in Microsporidia

**Figure 6:**
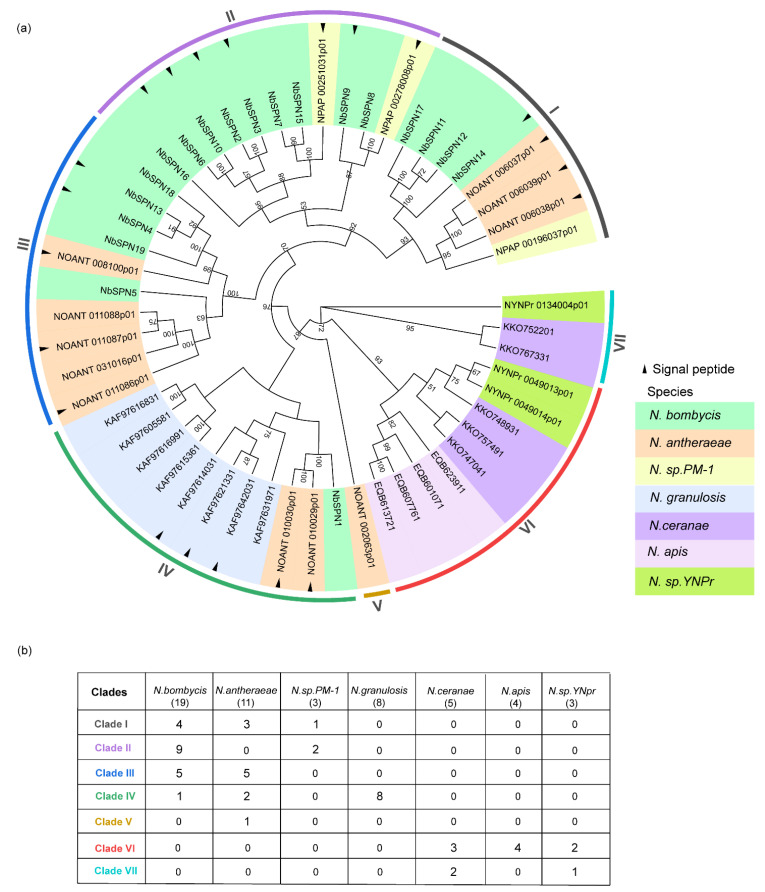
Phylogenetic analysis of serpin genes in microsporidia. (**a**) Different colors represent different species of serpins. The neighbor-joining phylogenetic tree was based on serpin protein sequences using MEGA-X. The analysis was conducted on 1000 bootstrapped datasets. Serpins with the predicted signal peptides (SignalP-5.0 Online: https://services.healthtech.dtu.dk/service.php?SignalP-5.0, accessed on 4 October 2022) were labeled with a black triangle. (**b**) The table records each species’ serpin numbers in seven clades in detail.

We screened the genomic data of over 50 species of microsporidia in the NCBI database, MicrosporidiaDB database (https://microsporidiadb.org/micro/app/, accessed on 10 June 2022), and SilkPathDB—Silkworm Pathogen Database (https://silkpathdb.swu.edu.cn/, accessed on 10 June 2022). Currently, all 53 identified serpins have only been found in the genus *Nosema*, which mainly infect insects and crustaceans. In addition to the 19 serpins in *N. bombycis*, a total of 34 serpins have been identified in the six genomes of *N. antheraeae* (11), *N. granulosis* (8), *Nosema sp. YNPr* (3), *Nosema sp*. PM-1 (3), *N. apis* (4), and *N. ceranae* (5) (Table S4). Thirteen out of 34 serpins had predicted signal peptides. The sizes of these 34 serpins varied from 187 Aa to 568 Aa, the average amino acid number was 372 Aa. There were six serpins with less than 300 Aa. Two pairs of tandem duplications were observed in *N. antheraeae*. Three serpins, *NOANT 006037*, *NOANT 006038*, *NOANT 006039,* were distributed in tandem and shared 100% percent identity; two other serpins, *NOANT 010029* and *NOANT 010030,* were also arranged in tandem, and these two serpins shared a 99.35% percent identity. To explore the evolutionary relationships of the serpin proteins in microsporidia, all 53 serpins from *Nosema* were selected to construct the phylogenetic tree (Figure 6). The tree shows that the *Nosema* serpins can be divided into seven clades. NbSPNs were mainly distributed in Clades I, II, III, and only NbSPN1 clustered in Clade IV. The serpins of *N. antheraeae* were distributed in Clades I, III, IV, and V. Notably, all eight serpins of *N. granulosis* were clustered together only in Clade IV, and the sequence percent identity among them varied from 24.22% to 85.04% All twelve serpins from *N. apis*, *N. ceranae,* and *Nosema* sp. *YNPr* gathered in Clades VI and VII. Interestingly, there was no signal peptide predicted in all of the serpins from these three species.

### 2.6. Phylogenetic Analysis to Determine the Evolution Position of the N. bombycis Serpins

**Figure 7:**
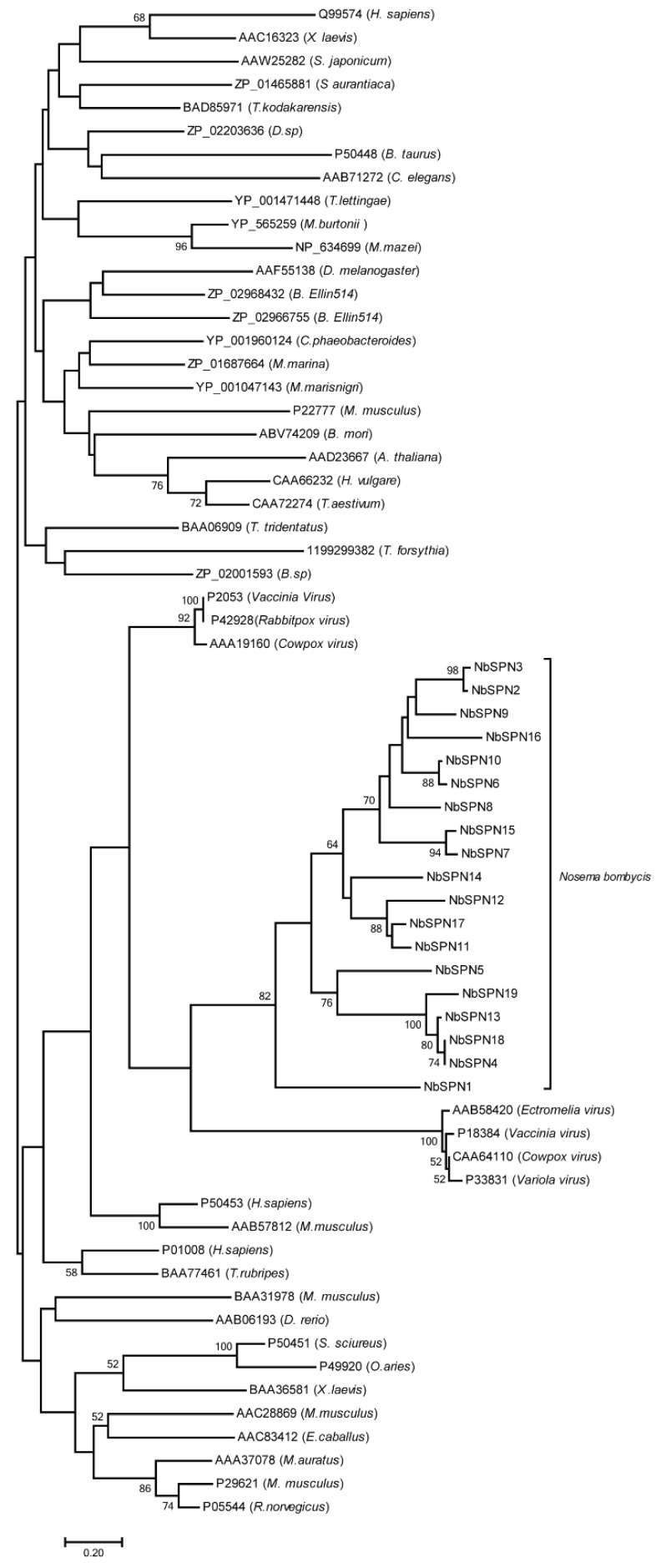
Phylogenetic tree of the serpin superfamily. The phylogenetic analysis of the serpins from *N. bombycis*, plants, animals, and viruses, etc., was from the NCBI GenBank database. The classification reference was from James A. Irving [34]. The end of each branch represents the GenBank accession number, and the name of the species is in parentheses. The phylogenetic analysis was performed using the MEGA-X neighbor-joining method. The analysis was performed on 1000 bootstrapped datasets.

To analyze the phylogeny of NbSPNs in all serpin superfamilies, we constructed a phylogenetic tree containing the serpin sequences of the animal, plant, bacteria, archaea, and virus from the NCBI database (Figure 7). The phylogenetic analysis showed that the NbSPNs clustered individually into one group and close to the branch of serpins from the *Chordopoxvirinae*, the subfamily of Poxvirus, which is mostly found in vertebrates. The phylogenetic tree showed that the evolution of NbSPNs was conservative and independent, and we do not yet have solid phylogenetic proof to conclude whether the origin of the *NbSPN* gene is horizontal gene transfer from viruses or its host.

### 2.7. Expression Profiles of NbSPNs in Infected BmE-SWU1 Cells

**Figure 8:**
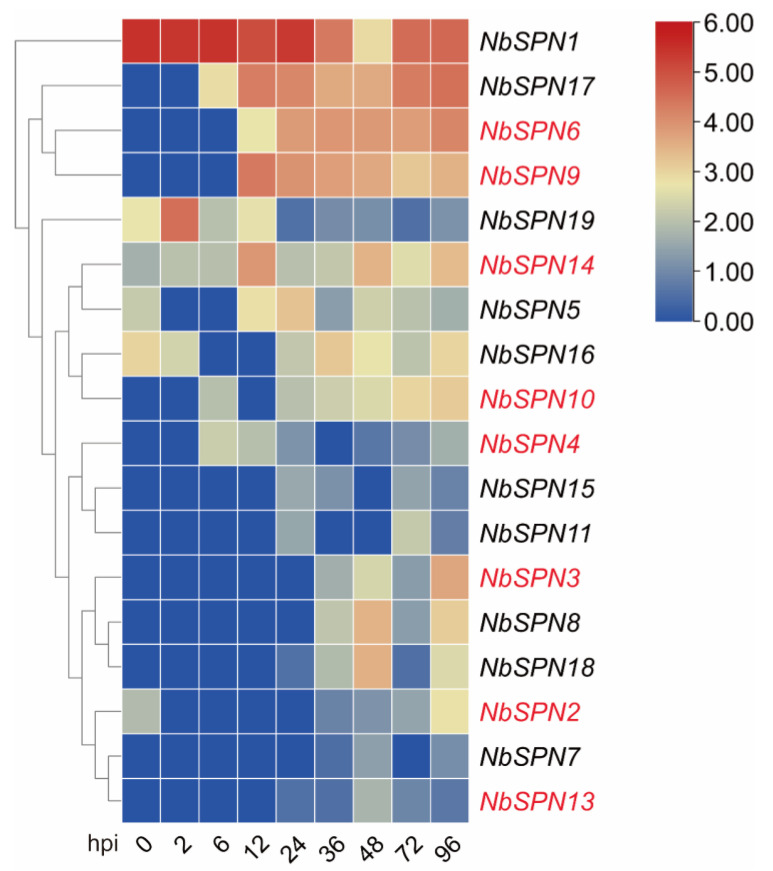
Expression analysis of NbSPNs in BmE-SWU1 cell line infected by *N. bombycis*. Heatmap generated by TBtools showing a cluster map of the NbSPN genes in infected BmE-SWU1 cells. The predicted genes that could encode signal peptides are marked in red. The color gradient (red/white/blue) indicates the gene expression level (high to low).

We analyzed the *NbSPN* expression at different time points between 0 and 96 h in the BmE-SWU1 (silkworm embryo) cell line after the infection of *N. bombycis* (Figure 8). The results showed that the NbSPN expression patterns were different. *NbSPN1* is highly transcribed during infection and proliferation. After infection for 6 h, the transcription of *NbSPN17*, *6*, and *9* began to increase continuously. Interestingly, *NbSPN19* was highly transcribed at the early stage of infection (2 hpi) and was downregulated at the later stage. The remaining genes maintained relatively low expression levels during the infection. The *NbSPN12* with the shortest sequence length was not recognized in the whole RNA-Seq data.

### 2.8. Expression Profiles of NbSPN Genes in Infected Silkworm Tissues

We investigated the transcriptional levels of the entire serpin family in the midgut and fat body of infected silkworm larvae using real-time fluorescence quantitative PCR. At least six infected silkworm midguts and fat bodies were randomly collected together as a sample pool at 1–6 days post-infection. Optical microscopy was used to detect mature spores in the silkworm’s midgut and fat body, confirming the successful infection of *N. bombycis*. Mature spores were observed in the midgut two days post-infection. As the days after infection increased, more mature spores in the midgut were observed. Quite a few mature spores were observed in the fat body four days post-infection (Figure S1).

**Figure 9:**
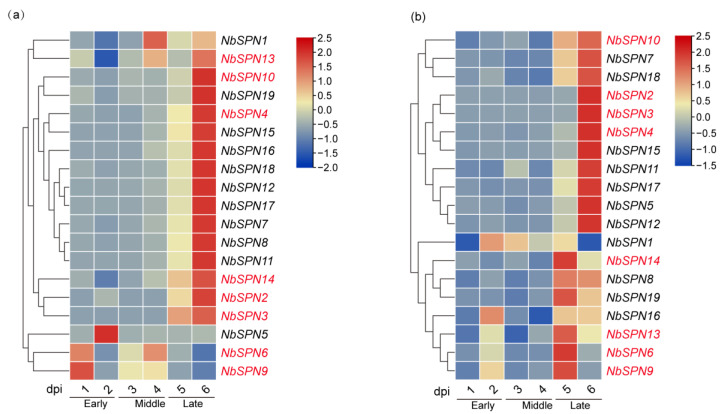
*N. bombycis* serpins’ transcription patterns of the midgut and the fat body in infected silkworms. They are divided into the early, middle, and late stages along with the time of infection. The predicted genes that could encode signal peptides are marked in red. (**a**) Transcriptional expression patterns of different serpin genes are in the infected midgut. (**b**) Transcriptional expression patterns of varying serpin genes are in the infected fat body.

In this study, real-time quantitative PCR analysis of *NbSPN* transcription patterns in the midgut of silkworms infected with *N. bombycis* was performed. The gene transcription in the infected midgut was mainly divided into two patterns (Figure 9). Most of the *NbSPN* gene transcription increased with the infection time, while the transcription characteristics of *NbSPN1*, *13*, *5*, *6*, *9* were different. The high transcription of *NbSPN1*, *13* occurred at four days post-infection. *NbSPN5*, *6*, *9* exhibited relatively high transcription in the early and middle stages of infection, but low in the late stage (Figure 9a). In the fat body infected by *N. bombycis*, the general expression pattern of NbSPNs reached a high level at 5 dpi or 6 dpi. Eleven *NbSPNs* (*NbSPN10*, *7*, *18*, *2*, *3*, *4*, *15*, *11*, *17*, *5*, *12*) shared a similar expression pattern where a higher level occurred at 6 dpi. The other seven *NbSPNs (NbSPN14*, *8*, *19*, *16*, *13*, *6*, *9*) achieved a high level at 5 dpi. Interestingly, the transcription of NbSPN1 was different to the others, which exhibited a high level at 2 dpi, but the lowest level at 6 dpi (Figure 9b). In general, most *NbSPNs* showed an increased expression level with infection time in the midgut and fat body; interestingly, compared with other *NbSPNs*, *NbSPN1* had a fairly unique expression feature in both infected tissues, which suggests a different role during the process of infection.

## 3. Discussion

Serpins are a class of widely distributed superfamily of protease inhibitors found in virus, prokaryotes, and eukaryotes [32]. Here, we identified 53 serpins in the *Nosema* genus of microsporidia including *N. bombycis*, *N. granulosis*, *N. antheraeae*, *Nosema* sp. *YNPr*, *Nosema* sp. PM-1, *N. apis*, and *N. ceranae*. In microsporidia, *N. bombycis* had the biggest serpin family with 19 members, followed by *N. antheraeae* with 11 members, both *Nosema* sp. *YNPr* and *Nosema* sp. PM-1 had the smallest serpin family with only three members. Twenty-one out of 53 serpins had the predicted signal peptides. The typical serpins of microsporidia included five motifs. Microsporidia serpin genes underwent duplication in the evolution and resulted in gene expansion in some species of *Nosema*. In *N. antheraeae* serpins, the *NOANT_006037*,*38*,*39* and *NOANT_010028*,*29* genes were shown as tandem duplications, respectively. In *N. bombycis,* NbSPN2,3, NbSPN4,13,18, NbSPN6,10, and NbSPN7,15 shared a high amino acid sequence identity, respectively, which also suggested that gene duplication occurred. Phylogenetic analysis showed that serpin duplication events in *N. bombycis* and *N. antheraeae* may occur before species differentiation, while duplication of serpin genes in *N. granulesis* may occur after species differentiation because all eight serpins were clustered in Clade IV (Figure 6). Gene duplication and differentiation are considered as the driving force for genes to produce new functions [35,36]. These results suggest that the microsporidia serpin family facilitates species of *Nosema* to adapt to the complex parasitic environment of the host through gene duplication.

Microsporidian genome evolution is a highly dynamic process that has balanced constraint, reductive evolution, and genome expansion during adaptation to an extraordinarily successful obligate intracellular lifestyle [37,38,39]. In the analyses of gain and loss, expansion and contraction of the protein family during the evolution of microsporidian genomes, 62 protein families were gained and 38 families were expanded in *N. ceranae,* respectively. Among them, the serpin family of *N. ceranae* was gained and expanded to five members [37]. One of the main approaches to gain a protein family for species is horizontal gene transfer [40]. In microsporidia, the nucleotide transport proteins (NTT) acquired through horizontal gene transfer (HGT) have been proven [40]. In addition, HGT examples from the host to microsporidia also include multiple transposable elements, septin and purine nucleotide phosphorylase (PNPs) [41,42,43]. Although our current data are not sufficient to determine where, when, and how the serpin genes of microsporidia originated, one possibility is that the *Nosema* serpin family may have been gained through horizontal gene transfer from its host or coexisted in intracellular pathogens in the same host cell such as poxvirus. Notably, we found that the *N. bombycis* serpins are phylogenetically clustered with serpins from Chordopoxvirinae, the poxvirus of vertebrates. Poxviridae consists of Chordopoxvirinae and Entomopoxvirinae (EPV), where EPV can infect a variety of insects including *Lepidoptera*, *Coleoptera*, *Orthotera*, and *Diptera* [44]. Several serpins from Chordopoxvirinae have been identified to inhibit the host apoptosis and inflammation [25,26]. However, we have not gleaned serpin genes from the EPV, which share common insect hosts with parasites of *Nosema*. Novel clues of the origin of *Nosema* serpin might be present with more microsporidia and EPV genomes sequenced.

Twenty-one *Nosema* serpins with the predicted signal peptides were thought to be secreted. Secretory serpins or non-secretory serpins may have different spatial localization and their different roles in parasite proliferation and the pathogenesis of microsporidia. Previous studies have revealed that some of the secreted proteins such as *N. bombycis* hexokinase (NbHK) were believed to participate in interactions between the parasite and silkworm [45,46]. Eight NbSPNs were predicted with signal peptides, suggesting that they can be secreted into host cells and interact with the host target proteins. Previously, we verified that NbSPN6 could inhibit the hemolymph melanization process via binding to PPAE, a prophenoloxidase-activating enzyme of the silkworm. Transcription of the secreted NbSPNs was detected in the midgut and fat body of *B. mori* infected by *N. bombycis*, which suggests that the secreted serpins may be involved in regulating the host biological processes facilitating *N. bombycis* infection. In further studies, we will use subcellular localization and other approaches to systemically analyze the functions of these secreted NbSPNs.

The expression patterns of *NbSPNs* tend to be similar in the midgut and fat bodies of infected silkworms. Most *NbSPNs* are highly expressed in the later stages of infection. The expression pattern of *NbSPNs* in the infected cell lines was different to that of the infected silkworm tissues. Compared with other *NbSPNs*, *NbSPN1* had a fairly different expression character in various infection scenes, at the same time, we noticed that NbSPN1 also shared a relative far distance with other NbSPNs in phylogeny. Notably, serpins are also able to perform non-inhibitory functions [11]. For example, heat shock protein 47 (HSP47) serves as a chaperone [47]. Thyroxine-binding globulin (TBG)1 and cortisol-binding globulin (CBG)2 all belong to the serpin superfamily and act as a hormone transporter [48]. Whether any serpins serve as non-inhibitory functions in microsporidia need further exploration in the future.

## 4. Materials and Methods

### 4.1. Silkworms and N. bombycis

The silkworm strain Dazao used in this study was provided by the State Key Laboratory of Silkworm Genome Biology, Southwest University, Chongqing, China. *Nosema bombycis* CQ1 was isolated from the State Key Laboratory of Silkworm Genome Biology and preserved at the China Veterinary Culture Collection Center (accession no. CVCCl02059).

### 4.2. Sequence Database Mining

The database for the *N. bombycis* genome used in this study contained a 6-fold coverage by the State Key Laboratory of Silkworm Genome Biology with 11,155 reads, 4479 predicted genes, and 1517 EST sequences [41]. Nucleotide and amino acid sequences of serpin homologs of humans, yeast, poxvirus, archaea, and other organisms were downloaded from the NCBI database (https://www.ncbi.nlm.nih.gov//genbank/), accessed on 20 October 2021. Other microsporidian genome data were obtained from Microsporidia DB (http://microsporidiadb.org/micro/, accessed on 10 June 2022.) [49] and the SilkPathDB-Silkworm Pathogen Database [50] (https://silkpathdb.swu.edu.cn/, accessed on 10 June 2022).

FASTA formats for all serpin-related gene sequences were downloaded from the GenBank database and compared to genomic databases for *Nosema bombycis*, *Encephalitozoon cuniculi*, *Nosema locustae*, *Nosema apis*, *Nosema pernyi*, and *Nosema ceranae* using BLASTP. An E-value <10^−3^ was used as the search threshold to identify the homologous serpin genes of *N. bombycis* and other microsporidia. Finally, a BLASTN search was performed on the EST database of *N. bombycis* using the nucleotide sequences of the serpin genes. The search threshold was set to an E-value <10^−5^, with identities >30% and a matching length exceeding 100 bp; the presence of the SERPIN domain indicated evidence of serpins.

### 4.3. Chromosomal Location and Gene Structure Analysis

The chromosomal locations of the NbSPN gene family members were extracted from the gff3 file of the *N. bombycis* genome annotation. A map of the chromosomal gene distribution was constructed using TBtools software (version 1.1047) [51]. The conserved amino acid sequences of the proteins were analyzed online using MEME software version 5.5.0 (http://MEME-suite.org, accessed on 28 June 2022); the number of motifs was set to five, and the other parameters were set to their default values. The motif structures were drawn using TBtools [51].

### 4.4. Multiple Alignments and Phylogenetic Analysis

The serpin amino acid sequences were retrieved from the *N. bombycis* genome database using ClustalX for BLASTP multiple sequence alignments; some of the typical serpins have already been reported in the literature. Human α-antitrypsin was used as a template sequence to analyze the sequence characteristics of the *N. bombycis* serpins. A phylogenetic tree was constructed using the neighbor-joining method in MEGA X, Poisson correction, complete deletion, and a bootstrap value of 1000. The resulting phylogenetic tree was further processed with the online tool iTOL (https://itol.embl.de/, accessed on 14 July 2021). We used the Simple Ka/Ks Calculator function of Tbtools software (version 1.1047) to calculate the base replacement rate of *NbSPN* gene pairs [51].

### 4.5. Oral Infection of Silkworms by N. bombycis Spores

The silkworms were reared at 25 °C and maintained at a suitable humidity of approximately 70% until the fourth molting for infection experiments. Newly developed spores were isolated from the infected silkworm pupae and purified using discontinuous Percoll gradient centrifugation (16,000 rpm, 40 min). Purified spores were rinsed twice with sterilized double-distilled water and stored with antibiotics (penicillin-streptomycin solution, 100×, Beyotime, China) at 4 °C. To rule out contamination by other agents, purified spores were added to the cell culture medium; if no contamination was found at 48 h post-infection, the purified spores were suitable for oral infection. One hundred fourth-instar molted silkworm larvae were placed in a Petri dish without food to maintain hunger before infection. The spores were washed with distilled water and then suspended at 10^9^ spores/mL. One milliliter of spore suspension was applied evenly to the folium mori to feed the hungry silkworm. To maintain a long period of spore persistence in the midgut, larvae were reared for 8 h. The midgut and fat bodies of the larvae were collected at 1–6 day time points after infection.

### 4.6. Real-Time Quantitative PCR Analysis

RNA was extracted using TRIzol™Reagent (Invitrogen, Carlsbad, CA, USA) and purified with a Nucleo Spin H RNA Clean-Up Kit (MACHEREY-NAGEL, Duren, Germany). One microgram of RNA from each sample was reverse-transcribed into cDNA using the EvoScript Universal cDNA Master Kit (Roche, Basel, Switzerland). Quantitative primers for real-time quantitative PCR amplification were designed using Primer 5.0 and synthesized (Sangon Biotech (Chengdu, China) (Table S1). Real-time quantitative PCR was performed as follows: denaturation at 95 °C for 2 min, followed by 40 cycles of 95 °C for 10 s and 60 °C for 20 s (CFX96TM Real-Time System, Bio-Rad, Richmond, CA, USA) [52]. Genomic DNA was isolated from *N. bombycis* using TRIzol™ Reagent (Invitrogen, Carlsbad, CA, USA). The *β-tubulin* gene of *N. bombycis* (GenBankNo.EOB14994.2) was used to normalize the qPCR data in this study. The extracted genomic DNA was used as a template to amplify the *N. bombycis* and *β-tubulin* genes since the *N. bombycis* genome had almost no introns [30].

## 5. Conclusions

In this study, 19 members of the NbSPN gene family were characterized. A total of 34 serpins from another six species of the *Nosema* genus were gleaned from the microsporidia genomes sequenced. The phylogenetic analyses provide a possible hypothesis that serpins of the *Nosema* genus may be gained through horizontal gene transfer from the host or co-infected intracellular pathogens. The large members of the NbSPN family suggest that they have evolved to carry out different functions to support microsporidia adaptation to the intracellular parasitic life. Future endeavors should focus on the origin and the functions of serpins in microsporidia proliferation and interaction with the host.

## Figures and Tables

**Table 1:** Basic information of serpins in *N. bombycis*. ^1^ MW, molecular weight; ^2^ pI, isoelectric point; ^3^ Inhibitory, Prediction of inhibitory serpin by RCL hinge region length and sequence consensus mode, “+” represents inhibitory serpin; “−” represents non-inhibitory serpin. Signal peptide was predicted with SignalP-5.0 online: https://services.healthtech.dtu.dk/service.php?SignalP-5.0, accessed on 6 June 2022.

Gene Name	Gene ID in SilkPathDB	GenBank Accession	Protein Length (Aa)	Signal Peptide (Aa)	MW ^1^ (kDa)	pI ^2^	Inhibitory ^3^
NbSPN1	NBO_13g0021	EOB14844	349	none	40.82	8.59	+
NbSPN2	NBO_18g0004	EOB14677	405	1–23	47.96	7.87	+
NbSPN3	NBO_18g0021	EOB14694	405	1–23	47.88	8.80	+
NbSPN4	NBO_19g0007	EOB14654	390	1–20	45.04	8.62	+
NbSPN5	NBO_19g0009	EOB14656	350	none	41.22	7.79	+
NbSPN6	NBO_34g0030	EOB14180	395	1–19	46.98	5.79	+
NbSPN7	NBO_34g0036	EOB14186	295	none	34.86	6.60	+
NbSPN8	NBO_35g0002	EOB14148	145	none	16.78	5.93	+
NbSPN9	NBO_39gi001	-	392	1–27	46.05	6.71	+
NbSPN10	NBO_42g0006	EOB14016	395	1–19	46.88	6.56	+
NbSPN11	NBO_44g0004	EOB13986	168	none	19.61	5.57	−
NbSPN12	NBO_44g0006	EOB13988	109	none	12.98	5.07	−
NbSPN13	NBO_124g0001	EOB13192	390	1–25	45.12	6.69	+
NbSPN14	NBO_372g0002	-	384	1–19	44.89	5.65	+
NbSPN15	NBO_1038g0001	EOB11591	132	none	15.23	4.47	+
NbSPN16	NBO_1125g0001	EOB11493	292	none	34.57	6.13	+
NbSPN17	NBO_1147g0001	EOB11479	206	none	24.17	5.56	+
NbSPN18	NBO_1569g0001	EOB11151	390	none	45.28	8.58	+
NbSPN19	NBO_1570g0002	EOB11150	181	none	21.10	9.32	+

## Data Availability

Not applicable.

## Supplementary Materials

The following supporting information can be downloaded at: https://www.mdpi.com/article/10.3390/ijms24010550/s1.
