# Supplementary material for: Genome-Wide Characterization and Comparative Genomic Analysis of the Serpin Gene Family in Microsporidian Nosema bombycis
Source: Int J Mol Sci. 2022 Dec 29;24(1):550. doi: 10.3390/ijms24010550 (PMC9820262; doi:10.3390/ijms24010550)
Supplement: Supplementary file 1 [file ijms-24-00550-s001.zip › FigureS1.Observation of midgut and fat body of N. bombycis oral-infected silkworms.pdf]

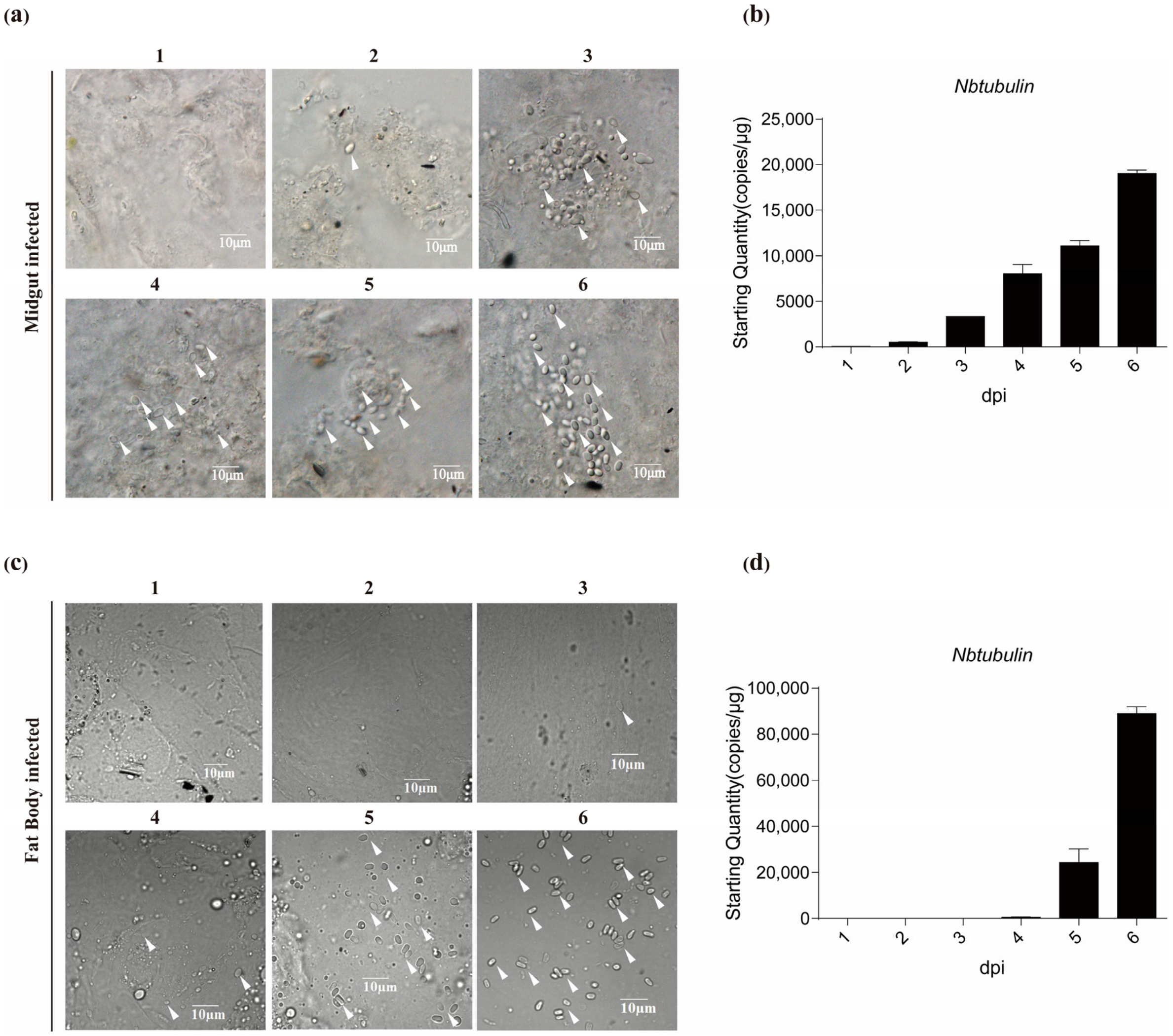

Figure S1. Observation of midgut and fat body of *N. bombycis* oral-infected silkworms. **(a)** Observation of spores in the midgut at different stages. **(b)** *N. bombycis* housekeeping gene *β-Tubulin* transcription pattern in the midgut after infection. **(c)** Observation of spores in the fat body at different stages. **(d)** *N. bombycis* housekeeping gene *β-Tubulin* transcription pattern in the fat body after infection. dpi: days post-infection.
